# LINC00461/miR-4478/E2F1 feedback loop promotes non-small cell lung cancer cell proliferation and migration

**DOI:** 10.1042/BSR20191345

**Published:** 2020-02-25

**Authors:** Qingxin Meng, Ming Liu, Ruyi Cheng

**Affiliations:** 1 Chest Surgery, Gansu Provincial Hospital of TCM, 518 Guazhou Road, Qilihe District, Lanzhou City 730050, Gansu Province, China; 2 Hand and Foot Orthopaedics, Gansu Provincial Hospital of TCM, 518 Guazhou Road, Qilihe District, Lanzhou City 730050, Gansu Province, China

**Keywords:** E2F1, LINC00461, miR-4478, non-small cell lung cancer

## Abstract

Non-small cell lung cancer (NSCLC) is a prevalent subtype of lung cancer, whose mortality is high. Long non-coding RNAs (lncRNAs) have caught rising attentions because of their intricate roles in regulating cancerization and cancer progression. Long intergenic non-protein coding RNA 461 (LINC00461) has recently shown oncogenic potential in several cancers, but the function of LINC00461 in NSCLC remains to be investigated. Our study planned to unveil the regulatory role of LINC00461 in NSCLC. It was validated that LINC00461 was highly expressed in NSCLC tissues and cell lines and exhibited prognostic significance. Furthermore, LINC00461 expression in advanced stage was much higher than in early stage. Loss-of-function experiments suggested that LINC00461 knockdown impaired cell proliferation, migration, and epithelial-to-mesenchymal transition (EMT). Subcellular fractionation revealed the predominant location of LINC00461 in cytoplasm. Mechanistically, LINC00461 up-regulated E2F transcription factor 1 (E2F1) expression through sponging miR-4478. Besides, E2F1 bound to the promoter of LINC00461 to induce its transcription. Finally, rescue experiments verified that LINC00461 aggravated proliferation, migration, and EMT through targeting miR-4478/E2F1 axis. In consequence, the present study illustrated that LINC00461/miR-4478/E2F1 feedback loop promoted NSCLC cell proliferation and migration, providing a new prognostic marker for NSCLC.

## Introduction

Non-small cell lung cancer (NSCLC), taking up approximately 80–85% of lung cancer cases, is a main histological subtype of lung cancer, and can be further classified into adenocarcinoma and squamous cell carcinoma [1,2]. Although some NSCLC patients promisingly benefit from early diagnosis and surgical tumor dissection, most patients remain disappointed by poor outcome with 20% five year survival rate [3,4]. Thus, further exploration on the molecular mechanism underlying NSCLC is of paramount significance for therapy development of NSCLC [5–7].

Long non-coding RNAs (lncRNAs) are defined as non-coding transcripts more than 200 nucleotides in length, with none or limited protein-coding potential [8,9]. Many lncRNAs have been shown to modulate a wide range of biological behaviors in tumors, such as autophagy, apoptosis, proliferation, invasion, and migration [10–13]. The effect of lncRNAs on NSCLC has also been uncovered by mounting researches [14–16]. Long intergenic non-protein coding RNA 461 (LINC00461) demonstrated oncogenic performance in breast cancer, myeloma, and glioma [17–19], promoting cell growth, migration, and invasion, but it is not researched in NSCLC.

Mechanistically, lncRNAs could realize their regulatory function through competing endogenous RNA (ceRNA) network in cytoplasm. Through sponging microRNAs (miRNAs), lncRNAs released downstream mRNAs which targeted by miRNAs [20]. miRNAs, known as small non-coding RNAs with approximately 22 nucleotides, are axiomatically reported to be gene repressors by binding to the 3′ untranslated region (3′UTR) so as to degrade mRNA or suppress translation [21]. Many miRNAs have been documented to exert repressive function in cancers [22,23], including NSCLC [24]. MiR-4478 is newly identified to present down-regulated expression in colorectal cancer [25,26], but it has rarely been researched in NSCLC.

E2F transcription factor 1 (E2F1) is reputed as a key transcription factor and regulates cell-cycle progression in cancers [27]. Besides, evidence has proved that E2F1 related to cancer metastasis [28,29]. Dysregulation of E2F1 has been demonstrated in a number of cancers, including lung cancer [30–32]. Importantly, E2F1 has been noted to activate oncogenes to facilitate tumor progression, and its activation on lncRNAs has been documented in colon cancer [33].

The present study was proposed to probe the role and molecular mechanism of LINC00461 in NSCLC, and it was discovered that LINC00461 exhibited a high expression level in NSCLC. Additionally, LINC00461 mediated by E2F1 facilitated NSCLC cell proliferation and migration through targeting miR-4478/E2F1 axis, revealing LINC00461/miR-4478/E2F1 feedback loop in NSCLC.

## Materials and methods

### Tissue specimens

Ninety paired cancer and adjacent para-cancerous tissues were collected from Gansu Provincial Hospital of TCM. All enrolled patients had signed written informed consent. The study was permitted by the Institutional Ethics Committee of Gansu Provincial Hospital of TCM. All tissues were maintained in liquid nitrogen and stored under −80°C. No patients received any preoperative therapies. Following the directions of World Medical Association Declaration of Helsinki, this work has been carried out. The ethical approval ID number is AF/SC-07/02.0.

### Cell lines and cell culture

The human normal bronchial epithelial cell line 16HBE, human NSCLC cell lines A549, H1299 (Cell Bank of Type Culture Collection of the Chinese Academy of Sciences, Shanghai, China); H23 and SPC-A1 (Cell Biology of Shanghai Institute, Shanghai, China) were used. For cell culture, Dulbecco’s Modified Eagle’s Medium (Invitrogen, Carlsbad, CA, U.S.A.) were applied for 16HBE, RPMI-1640 Medium (Invitrogen) for H1299, H23, and SPC-A1 cells, and F-12K Medium (Invitrogen) for A549 cells. All above-mentioned mediums were supplemented with 10% fetal bovine serum (Invitrogen) and penicillin–streptomycin (Sigma, U.S.A.) in the moist incubator at 37°C with 5% CO_2_.

### Cell transfection

Small interfering RNAs (siRNAs) targeting LINC00461 (siLINC00461#1/2) and short hairpin RNAs (shRNAs) targeting LINC00461 (shLINC00461#1/2) were used to knock down LINC00461. The pcDNA3.1/LINC00461 or pcDNA3.1/E2F1 was used to overexpress LINC00461 or E2F1. siNC, shNC, and pcDNA3.1 vectors were controls. The microRNA 4478 (miR-4478) mimic and miR-4478 inhibitor were used for miR-4478 overexpression and knockdown, NC mimic and NC inhibitor as controls. Plasmids sequences used in the present study were listed as below:

siNC: CGAUGUUACAUAACUUAUUAG
siLINC00461#1: CGAUAAGUUAUGUAACAUUAG
siLINC00461#2: GUUAAUUGUAGUAGACAAUGG
shNC: CCGCCGATGTTACATAACTTATTAGCTCGAGCTAATAAGTTATGTAACATCGTTTTTG
shLINC00461#1: CCGCCGATAAGTTATGTAACATTAGCTCGAGCTAATGTTACATAACTTATCGTTTTTG
shLINC00461#2: CCGCGTTAATTGTAGTAGACAATGGCTCGAGCCATTGTCTACTACAATTAACTTTTTG
NC mimic: GUCAGCCUGCUGAGGAG
miR-4478 mimic: GAGGCUGAGCUGAGGAG
NC inhibitor: CUCCUCAGCAGGCUGAC
miR-4478 inhibitor: CUCCUCAGCUCAGCCUC

All vectors were produced by GenePhamar (Shanghai, China). The introduction of plasmids was accomplished by Lipofectamine 2000 (Invitrogen) as demanded, and cells were harvested 2 days after transfection.

### Quantitative real-time PCR

To obtain RNA extracts, TRIzol reagent (Invitrogen) was used. The complementary DNA (cDNA) was produced from extracted RNAs utilizing PrimeScript RT reagent Kit and First-Strand cDNA Synthesis Kit (GeneCopoeia, Guangzhou, China) with the genomic DNA (gDNA) Eraser kit (Takara, Dalian, China). The PCR reactions were accomplished utilizing SYBR Premix Ex Taq II (Takara) on a StepOne Plus Real Time PCR System (Life Technologies). Small nuclear RNA U6 (for miRNA) and glyceraldehyde-3-phosphate dehydrogenase (GAPDH) (for lncRNA and mRNA) were internal controls. Primers were as follows:

**Table:** 

LINC00461	5′-GACATTTACGCCACAACCCACG-3′
	5′-AGACAGACCCTCAGATTCCCCA-3′
E2F1	5′-CCCATCCCAGGAGGTCACTT-3′
	5′-CTGCAGGCTCACTGCTCTC-3′
MiR-4478	5′-AGGGCTAGGTGGAAAGACCT-3′
	5′-CCTTCCTGATCGGGACATCG-3′
GAPDH	5′-CCACATCGCTCAGACACCAT-3′
	5′-TGACAAGCTTCCCGTTCTCA-3′
U6	5′-CTCGCTTCGGCAGCACA-3′
	5′-AACGCTTCACGAATTTGCGT-3′

### Cell counting kit-8 assay

Cell counting kit-8 (CCK-8) cell counting kit (Zoman, Beijing, China) was applied for cell viability detection. Transfected cells were inoculated in 96-well plates (1000 cells/well). 10 μl CCK-8 solution was added at incubation time points at day 1, 2, 3, and 4. Following further incubation for another hour at 37°C, the optical density (OD) value (450 nm) was estimated under a microplate reader (EXL800 from BioTek, Winooski, Vermont, U.S.A.).

### Transwell migration assay

For detecting cell migration, transwell chambers (pore size at 8 μm) (corning, New York, U.S.A.) were applied. Non-serum medium with transfected cells (1 × 10^5^/well) was added to the upper insert, whereas 20% serum-contained medium was added into the lower insert. After 20 h incubation at 37°C, cells remained in upper chambers were scrubbed off by the cotton swab, and migrated cells on the bottom surface were fixed utilizing 4% PFA and stained utilizing 0.1% crystal violet. Stained cells were evaluated in five randomly chosen fields under the optical microscope.

### Dual-luciferase reporter assay

The pmirGLO Dual-Luciferase vectors were used (Promega) for luciferase reporter assay. The E2F1 3′UTR or LINC00461 containing miR-4478 binding sequences were inserted into the pmirGLO vectors to produce WT-LINC00461 and WT-E2F1. The Mut-LINC00461 and Mut-E2F1 (with mutated binding sites) were generated as well. MiR-4478 mimic or NC mimic were co-transfected with WT-LINC00461 or Mut-LINC00461, or WT-E2F1 or Mut-E2F1 into 293T cells (ATCC; Manassas, VA, U.S.A.) utilizing Lipofectamine 2000 (Invitrogen). The pGL3-LINC00461 promoter was co-transfected with pcDNA3.1/E2F1 or pcDNA3.1 into cells. 48 h following transfection, luciferase activity was assessed utilizing the Dual-Luciferase Reporter Assay System (Promega). Renilla luciferase activity was applied as normalized control.

### RNA pull-down

Briefly, LINC00461 biotin probe and LINC00461 non-biotin probe were generated by GenePharma (Shanghai, China). Then, LINC00461 biotin probe and LINC00461 non-biotin probe were incubated with cell lysate and streptavidin-coated magnetic beads (Ambion, Life Technologies). The abundance of candidate miRNAs in biotin-coupled RNA complex was tested by quantitative real-time PCR (RT-qPCR).

### Chromatin immunoprecipitation

To evaluate the binding of E2F1 on LINC00461 promoter, a ChIP Assay Kit (Millipore) was used. The extracted chromatin DNAs were sonicated into 200–400 bp fragments and subjected to immunoprecipitation with the antibodies against IgG (ab205719; Abcam, Cambridge, MA, U.S.A.) or E2F1 (ab179445; Abcam). The precipitated DNAs were analyzed by RT-qPCR.

### Western blot

The cell lysates were obtained by RIPA buffer (Keygen Biotech, Jiangsu, China) with 1% protease inhibitors (Thermo Scientific, Rockford, IL, U.S.A.). After separation on SDS-polyacrylamide gels (12%), the proteins were loaded onto polyvinylidene fluoride membranes (Millipore, Billerica, MA, U.S.A.) which were later sealed for 1 h with 5% skim milk. Subsequently, membranes went through overnight incubation with primary antibodies at 4°C and then 1 h incubation with secondary antibody (Santa Cruz Biotech, CA, U.S.A.). The following primary antibodies were used: anti-E2F1 (ab179445; Abcam, Cambridge, MA, U.S.A.), anti-N-cadherin (4370S; Cell Signaling Technology), anti-E-cadherin (4695S; Cell Signaling Technology, Beverly, MA, U.S.A.), anti-Vimentin (ab92547; Abcam), anti-Slug (ab27568; Abcam), anti-Twist (ab50887; Abcam), and anti-GAPDH (MB001; Bioworld Technology, St Louis Park, MN, U.S.A.).

### Cytoplasmic/nuclear fractionation

In accordance with the protocols provided by the suppliers, PARIS™ Kit (Invitrogen™, AM1921) was used for nuclear and cytoplasmic fractionation assay in NSCLC cells. NSCLC cells in fractionation buffer went through centrifugation, followed by collection of the supernatant. We washed the remaining lysates in fractionation buffer, and cell nuclei were obtained via cell disruption buffer. Thereafter, RT-qPCR was utilized to detect the cytoplasmic and nuclear LINC00461.

### Fluorescence *in situ* hybridization assay

Four percent paraformaldehyde was used to fix SPC-A1 and A549 cells maintained on glass slides, followed by being permeabilized with 0.5% TritonX-100. LINC00461 probe was utilized for hybridizing SPC-A1 and A549 cells by the ViewRNA™ ISH Cell Assay Kit (Thermo Fisher Scientific) referring to the manufacturer’s protocol, as described previously [34]. At last, Leica TCS-SL confocal microscope was used for sample visualization.

### Immunofluorescence staining

SPC-A1 and A549 cells were maintained in 6-well plates, and later transfected with shNC or shLINC00461#1/2 for 48 h. Cells were then fixed in 4% paraformaldehyde and incubated at 4°C with E-cadherin and N-cadherin primary antibodies overnight and anti-rabbit IgG which was fluorochrome-labeled. Prior to confocal microscopy analysis, DAPI was applied for staining cells.

### Statistical analysis

Data presentation was realized by mean ± S.D. Statistical analysis was accomplished on the SPSS 18.0 statistical software (IBM, New York, NY). Student’s *t*-test or one-way/two-way ANOVA analyses were used respectively for evaluating two-group or multi-group variances. All assays were triplicated. *P*<0.05 was used to define the significance of differences.

## Results

### LINC00461 was up-regulated in NSCLC and silenced LINC00461 inhibited NSCLC cell proliferation, migration, and EMT

**Figure 1:**
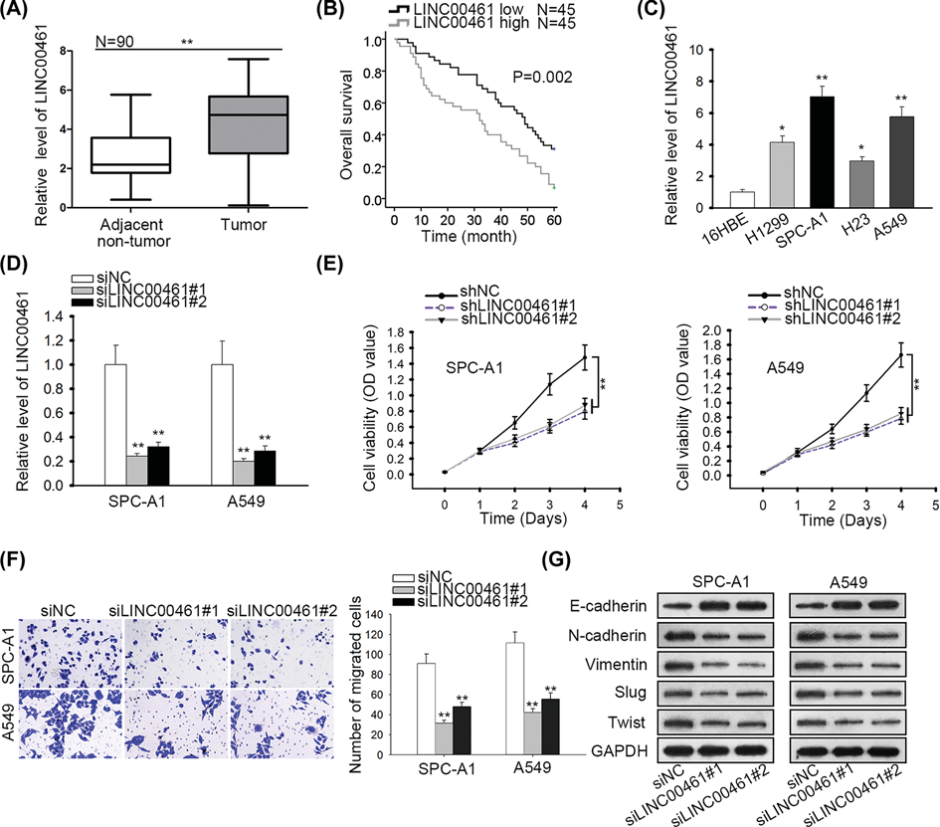
LINC00461 was up-regulated in NSCLC cells and silenced LINC00461 inhibited NSCLC cell proliferation, migration, and EMT (**A**) RT-qPCR showed LINC00461 expression in NSCLC tissues and matched non-cancerous tissues. (**B**) Kaplan–Meier analysis showed that high LINC00461 level indicated poor prognosis among NSCLC patients. (**C**) LINC00461 expression in NSCLC cell lines and 16HBE cell line was detected by RT-qPCR. (**D**) Transfection efficiency of siLINC00461 was determined by qRT-PCR in SPC-A1 and A549 cells. (**E**) Cell proliferation in response to shLINC00461 transfection was analyzed through CCK-8 assay. (**F**) Transwell assay manifested cell migration responding to LINC00461 silencing. (**G**) Western blot assay confirmed the protein levels of E-cadherin, N-cadherin, Vimentin, Slug, and Twist in cells transfected with siLINC00461. *P<0.05, **P<0.01.

First, we explored the expression level of LINC00461 in NSCLC tissues (*n*=90), and matched para-cancerous tissues (*n*=90) was taken as a reference. The results indicated that LINC00461 was up-regulated in NSCLC tissues (Figure 1A). Furthermore, high expression of LINC00461 suggested low overall survival in NSCLC patients (Figure 1B). The relevance of LINC00461 expression with tumor stage was also examined. It was illustrated that higher level of LINC00461 presented in III and IV stages (advanced stage) than in I and II stages (early stage) (Supplementary Figure S1A). Additionally, we detected LINC00461 expression in NSCLC cell lines (H1299, SPC-A1, H23, and A549) and human normal bronchial epithelial cell line (16HBE). As a result, LINC00461 expression was higher in NSCLC cell lines, especially in SPC-A1 and A549 cells, than in 16HBE cell line (Figure 1C). Thereafter, we investigated the biological function of LINC00461 in NSCLC. Using siRNAs or shRNAs targeting LINC00461, LINC00461 expression was knocked down in SPC-A1 and A549 cells (Figure 1D and Supplementary Figure S1B). Then, loss-of-function assays were carried out. Through CCK-8 assay, results manifested that the proliferation of SPC-A1 and A549 cells was attenuated by shLINC00461 transfection (Figure 1E). Besides, we detected the influence of silenced LINC00461 on cell migration via transwell assay. It was discovered that LINC00461 knockdown hampered the migratory ability of SPC-A1 and A549 cells (Figure 1F). To investigate the effect of LINC00461 on epithelial-to-mesenchymal transition (EMT), Western blot analysis was performed. The results indicated that the transfection of siLINC00461 resulted in an increased level of E-cadherin (epithelial marker) and a declined level of N-cadherin, Vimentin (mesenchymal markers), Slug and Twist (downstream oncogenic transcriptional activators) (Figure 1G). To further confirm the promotive role of LINC00461 in EMT process, immunofluorescence staining was conducted using shLINC00461. The results suggested that EMT process was retarded by LINC00461 deficiency (Supplementary Figure S1C). Therefore, above results suggested that LINC00461 was up-regulated in NSCLC and silenced LINC00461 hampered NSCLC cell proliferation, migration, and EMT.

### LINC00461 sponged miR-4478 in NSCLC cells

**Figure 2:**
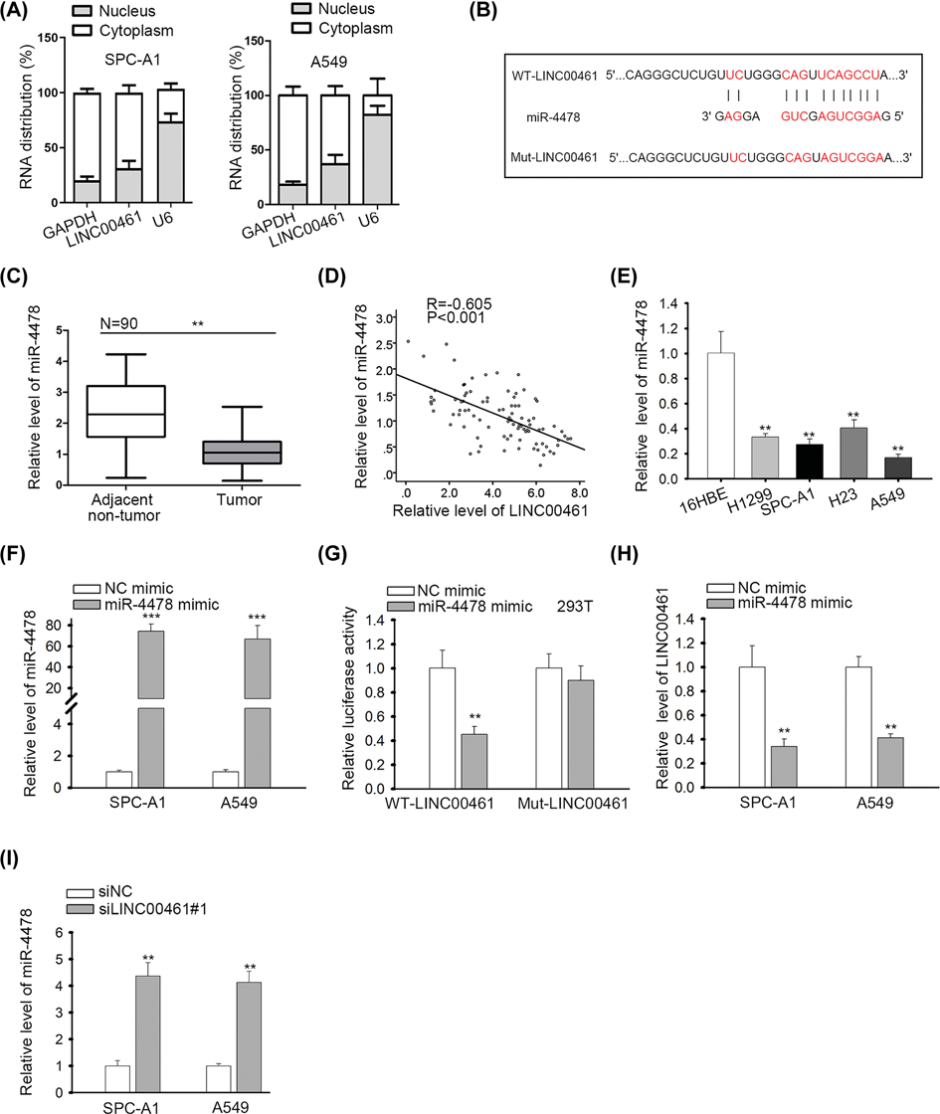
LINC00461 sponged miR-4478 in NSCLC cells (**A**) Cellular location of LINC00461 in SPC-A1 and A549 cells was analyzed by subcellular fractionation. (**B**) The binding site between LINC00461 and miR-4478 was predicted and the mutant site was constructed. (**C**) RT-qPCR was applied for detecting miR-4478 expression in NSCLC tissues and matched para-cancerous tissues. (**D**) Spearman’s correlation curve showed the negative relation between miR-4478 and LINC00461. (**E**) RT-qPCR exhibited miR-4478 expression in NSCLC cells. (**F**) Results of RT-qPCR confirmed the overexpression efficiency of miR-4478 mimic. (**G**) The luciferase reporter assay validated the interaction between LINC00461 and miR-4478. (**H**) Effect of miR-4478 mimic on LINC00461 expression was confirmed. (**I**) MiR-4478 expression in siLINC00461 transfected cells was detected by RT-qPCR. **P<0.01, ***P<0.001.

It was reported that the regulatory mechanism that lncRNAs mediated in cancer progression depended on their localization in cells [35]. To decipher the mechanism of LINC00461 in NSCLC, we performed subcellular fractionation assay. It was shown that LINC00461 was primarily expressed in cytoplasm (Figure 2A). Fluorescence *in situ* hybridization (FISH) assay further corroborated that LINC00461 was a cytoplasmic RNA (Supplementary Figure S1D). In cytoplasm, lncRNAs could act as a ceRNA through competing with downstream mRNAs for miRNAs [20]; therefore, we searched for the miRNAs that could bind to LINC00461 through LncBase (http://carolina.imis.athena-innovation.gr/diana_tools/web/index.php?r=lncbasev2%2Findex-predicted). Among those potential LINC00461-associated miRNAs, we chose the top 200 miRNAs. Subsequently, RT-qPCR examined differential expression of these miRNAs in 16HBE (normal control) and three NSCLC cells (SPC-A1, H1299, A549). Results of RT-qPCR confirmed eight down-regulated miRNAs in NSCLC (Supplementary Figure S2A). RNA pull-down assay demonstrated that miR-4478 was significantly enriched in biotinylated LINC00461 probe while other seven miRNAs did not present significant enrichment (Supplementary Figure S2B). Consequently, miR-4478 was identified as a downstream miRNA of LINC00461 in NSCLC. The binding site between LINC00461 and miR-4478 was depicted in Figure 2B. We validated that miR-4478 was pronouncedly down-regulated in NSCLC tissues (Figure 2C), and was negatively correlated with LINC00461 expression in NSCLC samples (Figure 2D). Additionally, miR-4478 presented a low expression level in NSCLC cell lines (Figure 2E). Next, we overexpressed miR-4478 by miR-4478 mimic in SPC-A1 and A549 cells (Figure 2F). Luciferase reporter assay confirmed that miR-4478 overexpression remarkably decreased the luciferase activity of WT-LINC00461 rather than Mut-LINC00461 (Figure 2G). Moreover, overexpression of miR-4478 reduced LINC00461 expression and knockdown of LINC00461 induced miR-4478 expression (Figure 2H,I). In conclusion, these results indicated that LINC00461 sponged miR-4478 in NSCLC cells.

### LINC00461/miR-4478/E2F1 formed a positive regulatory feedback loop in NSCLC cells

**Figure 3:**
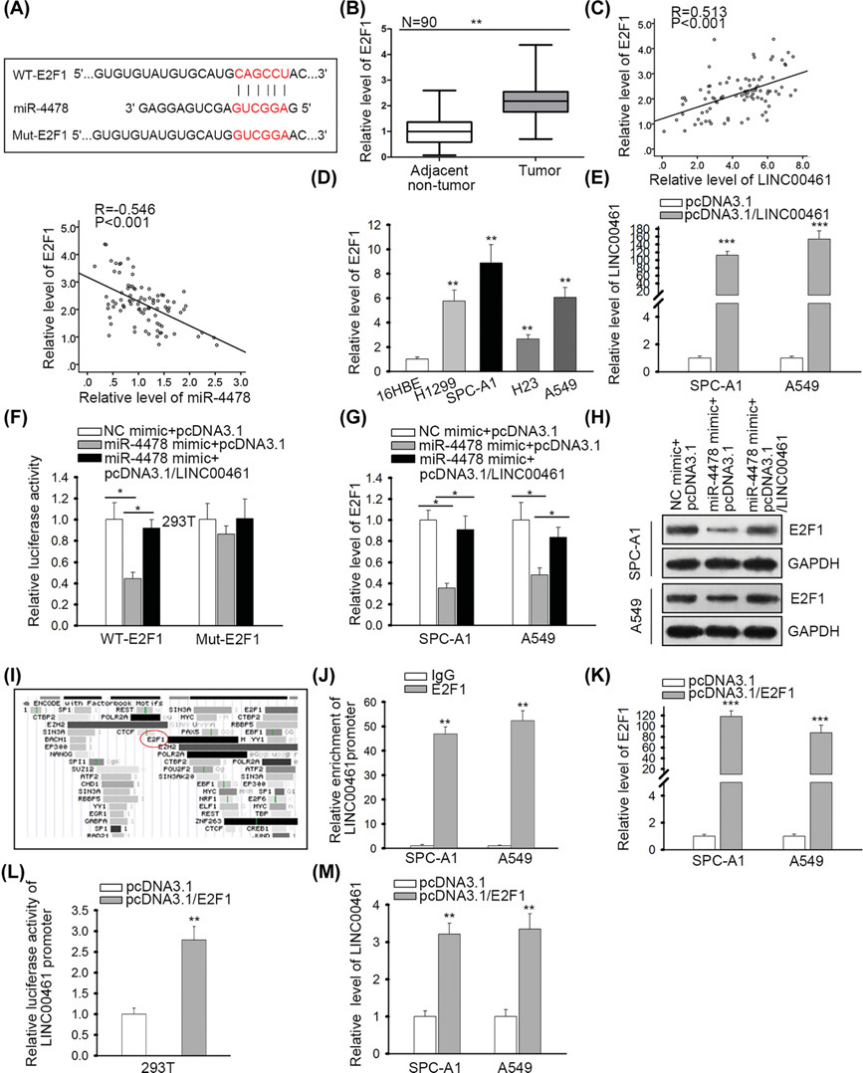
LINC00461/miR-4478/E2F1 formed a positive regulatory feedback loop in NSCLC cells (**A**) The wide-type or mutant binding site between E2F1 and miR-4478 was illustrated. (**B**) E2F1 expression in NSCLC tissues was assessed. (**C**) Spearman’s correlation analysis presented the correlation between E2F1 and LINC00461 or miR-4478. (**D**) RT-qPCR was employed to evaluate E2F1 expression in NSCLC cells. (**E**) Transfection efficiency of pcDNA3.1/LINC00461 was validated through RT-qPCR. (**F**) Luciferase reporter assay demonstrated the luciferase activity of WT-E2F1 or Mut-E2F1 in 293T cells transfected with NC mimic+pcDNA3.1, miR-4478 mimic+pcDNA3.1, or miR-4478 mimic+pcDNA3.1/LINC00461. (**G,H**) RT-qPCR and Western blot assays indicated E2F1 mRNA expression and protein level in each group. (**I**) E2F1 was predicted as a potential transcription factor at LINC00461 promoter via UCSC database. (**J**) ChIP confirmed the binding of E2F1 to LINC00461 promoter. (**K**) Results of RT-qPCR unveiled the overexpression of E2F1 caused by pcDNA3.1/E2F1. (**L**) Luciferase reporter assay validated the interaction between LINC00461 promoter and E2F1. (**M**) Results of RT-qPCR implied the effect of E2F1 on LINC00461 expression. *P<0.05, **P<0.01, ***P<0.001.

Subsequently, we searched for the target mRNAs through TarBase (http://carolina.imis.athena-innovation.gr/diana_tools/web/index.php?r=tarbasev8%2Findex). It was discovered that E2F1 was a putative target for miR-4478. E2F1 has been documented to present a high expression and could aggravate cell proliferation, invasion, and metastasis in NSCLC [30–32]. Thus, we further investigated E2F1 in NSCLC. The binding site and mutant site of E2F1 on miR-4478 were shown in Figure 3A. RT-qPCR validated that E2F1 was highly expressed in NSCLC tissues (Figure 3B). Moreover, E2F1 was confirmed to be positively correlated with LINC00461 and negatively associated with miR-4478 (Figure 3C). Furthermore, E2F1 exhibited higher expression in NSCLC cell lines (Figure 3D). To determine whether E2F1 was involved in LINC00461-mediated ceRNA network, we overexpressed LINC00461 in NSCLC cells (Figure 3E). Then, luciferase reporter assay illustrated that the luciferase activity of WT-E2F1 reduced by overexpressed miR-4478 could be reversed upon LINC00461 overexpression but that of Mut-E2F1 was not affected (Figure 3F). Besides, RT-qPCR and Western blot analysis demonstrated that up-regulated LINC00461 restored miR-4478 overexpression-mediated suppression on E2F1 mRNA expression and protein level (Figure 3G,H). All the results suggested that LINC00461 functioned as a ceRNA in NSCLC by targeting miR-4478/E2F1 axis.

Recent researches showed that lncRNAs could form a positive feedback loop with their target genes in ceRNA network [36,37]. Since E2F1 was identified as a transcription activator [38,39], we wondered whether E2F1 could transcriptionally activate LINC00461 expression. Using UCSC (http://genome.ucsc.edu/), we found that E2F1 was a potential transcription factor at LINC00461 promoter (Figure 3I). To confirm this, chromatin immunoprecipitation (ChIP) assay was conducted. Results revealed the abundant enrichment of LINC00461 promoter in E2F1 precipitates (Figure 3J). To further probe the interaction between E2F1 and LINC00461 promoter, we overexpressed E2F1 in NSCLC cells (Figure 3K). Luciferase reporter assay demonstrated an enhanced luciferase activity of LINC00461 promoter reporter in response to E2F1 overexpression (Figure 3L). Additionally, overexpression of E2F1 induced LINC00461 expression in NSCLC cells (Figure 3M). These results indicated that LINC00461/miR-4478/E2F1 formed a positive regulatory feedback loop in NSCLC.

### LINC00461/miR-4478/E2F1 axis regulated NSCLC cell proliferation, migration, and EMT

**Figure 4:**
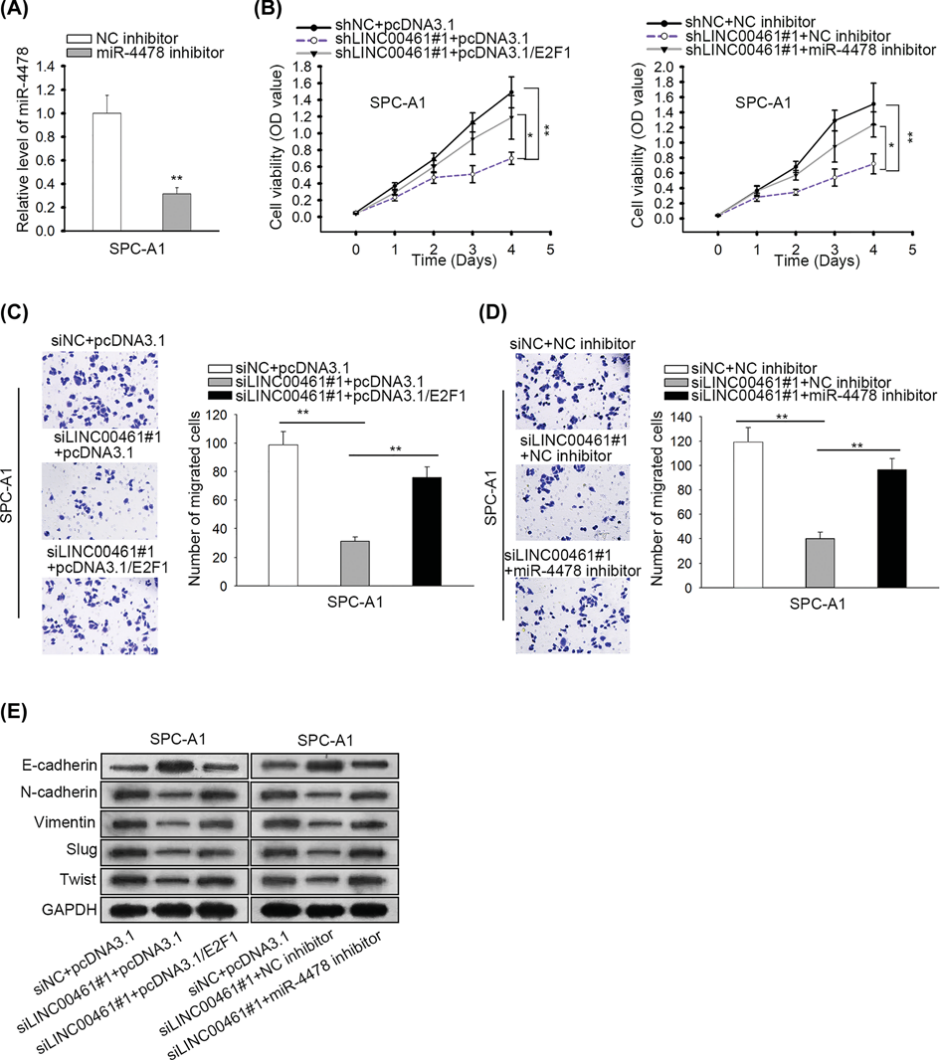
LINC00461/miR-4478/E2F1 axis regulated NSCLC cell proliferation, migration, and EMT (**A**) RT-qPCR confirmed the knockdown of miR-4478 by miR-4478 inhibitor. (**B**) CCK-8 was used to estimate cell proliferation in each group. (**C,D**) Transwell migration assay showed cell migration in each group. (**E**) EMT process in cells transfected with appointed plasmids was analyzed by Western blot assay. *P<0.05, **P<0.01.

To inquire the role of LINC00461/miR-4478/E2F1 axis in NSCLC, we performed rescue experiments. Before the experiments, the inhibition of miR-4478 in SPC-A1 was determined by RT-qPCR (Figure 4A). According to CCK-8 assay, we observed that E2F1 overexpressing or miR-4478 inhibition restored the attenuated proliferation caused by LINC00461 silencing (Figure 4B). Then, transwell assay demonstrated that cell migration hindered by LINC00461 deficiency could be abolished with the transfection of pcDNA3.1/E2F1 or miR-4478 inhibitor (Figure 4C,D). Results of Western blot assay validated that the accelerated EMT process in LINC00461 silenced cells was impaired by E2F1 overexpression or miR-4478 suppression (Figure 4E). Taken together, LINC00461/miR-4478/E2F1 axis promoted NSCLC cell proliferation, migration, and EMT.

## Discussion

Mounting studies have suggested the significant role of lncRNAs in the development of cancers, including NSCLC [14–16]. Herein, we selected LINC00461 for investigation because it has been demonstrated as an oncogene in multiple cancers, such as breast cancer, myeloma, and glioma [17–19]. In the present study, we firstly found that LINC00461 was up-regulated in NSCLC, and indicated poor prognosis. Additionally, LINC00461 was highly expressed in late stages. Functionally, LINC00461 silencing hampered proliferation, migration, and EMT in NSCLC cell lines. These results proved the oncogenic property of LINC00461 in NSCLC.

Former reports suggested that lncRNAs can regulate cancer progression via ceRNA network in cytoplasm, whereby lncRNAs could prevent miRNAs from targeting downstream messenger RNAs (mRNAs) by acting as miRNA sponges [20]. In our study, we confirmed the predominant expression of LINC00461 in cytoplasm, indicating that LINC00461 could potentially act as a ceRNA. Then, through bioinformatics tools, we found that miR-4478 was a candidate target miRNA for LINC00461. Previous studies showed that miR-4478 was down-regulated in colorectal cancer [25,26]. In concordance, we confirmed the down-regulation of miR-4478 in NSCLC tissues and cells, and revealed the negative expression correlation between miR-4478 and LINC00461. Thereafter, LINC00461 was proved to interact with miR-4478 in NSCLC. To be concluded, LINC00461 acted as a sponge of miR-4478 in NSCLC.

E2F1 is defined as a transcription activator and reported to regulate cell cycle, cell growth, and metastasis in cancers [27–29]. The oncogenic function of E2F1 in NSCLC was also documented [30–32]. In the present research, we identified the up-regulation of E2F1 in NSCLC tissues and cell lines. Besides, E2F1 showed a positive expression association with LINC00461 and negative expression relevance to miR-4478. Moreover, E2F1 was verified as a target gene for miR-4478. Former study revealed that E2F1 could promote cancer progression through transcriptionally activating some genes [38,39]. Importantly, the transcriptional of E2F1 on lncRNA has been demonstrated in colon cancer [33]. Interestingly, E2F1 was also identified as a transcription factor at LINC00461 promoter through UCSC database. We further testified the interaction of E2F1 with LINC00461 promoter and its promotive effect on LINC00461 expression, suggesting that LINC00461 could be transcriptionally activated by E2F1 in NSCLC. Finally, rescue assays consolidated the role of LINC00461/miR-4478/E2F1 in NSCLC.

To be concluded, E2F1-activated LINC00461 functioned as an oncogene in NSCLC. In addition, LINC00461 predicted a poor prognosis and promoted NSCLC proliferation, migration, and EMT. All results showed LINC00461/miR-4478/E2F1 positive feedback loop in NSCLC, indicating that LINC00461 could be a new prognostic marker. This finding provided a theoretic basis to explore the promising therapeutic strategies for NSCLC patients.

## Supplementary Material

Supplementary Figures S1 and S2

## Abbreviations

CCK-8: Cell counting kit-8
E2F1: E2F transcription factor 1
EMT: epithelial-to-mesenchymal transition
FISH: fluorescence *in situ* hybridization
GAPDH: glyceraldehyde-3-phosphate dehydrogenase
lncRNA: long non-coding RNA
NSCLC: non-small cell lung cancer
RT-qPCR: quantitative real-time PCR
