# Supplementary material for: LINC00461/miR-4478/E2F1 feedback loop promotes non-small cell lung cancer cell proliferation and migration
Source: Biosci Rep. 2020 Feb 25;40(2):BSR20191345. doi: 10.1042/BSR20191345 (PMC7042125; doi:10.1042/BSR20191345)
Supplement: Supplementary Figures S1 and S2 [file BSR-2019-1345_supp.pdf]

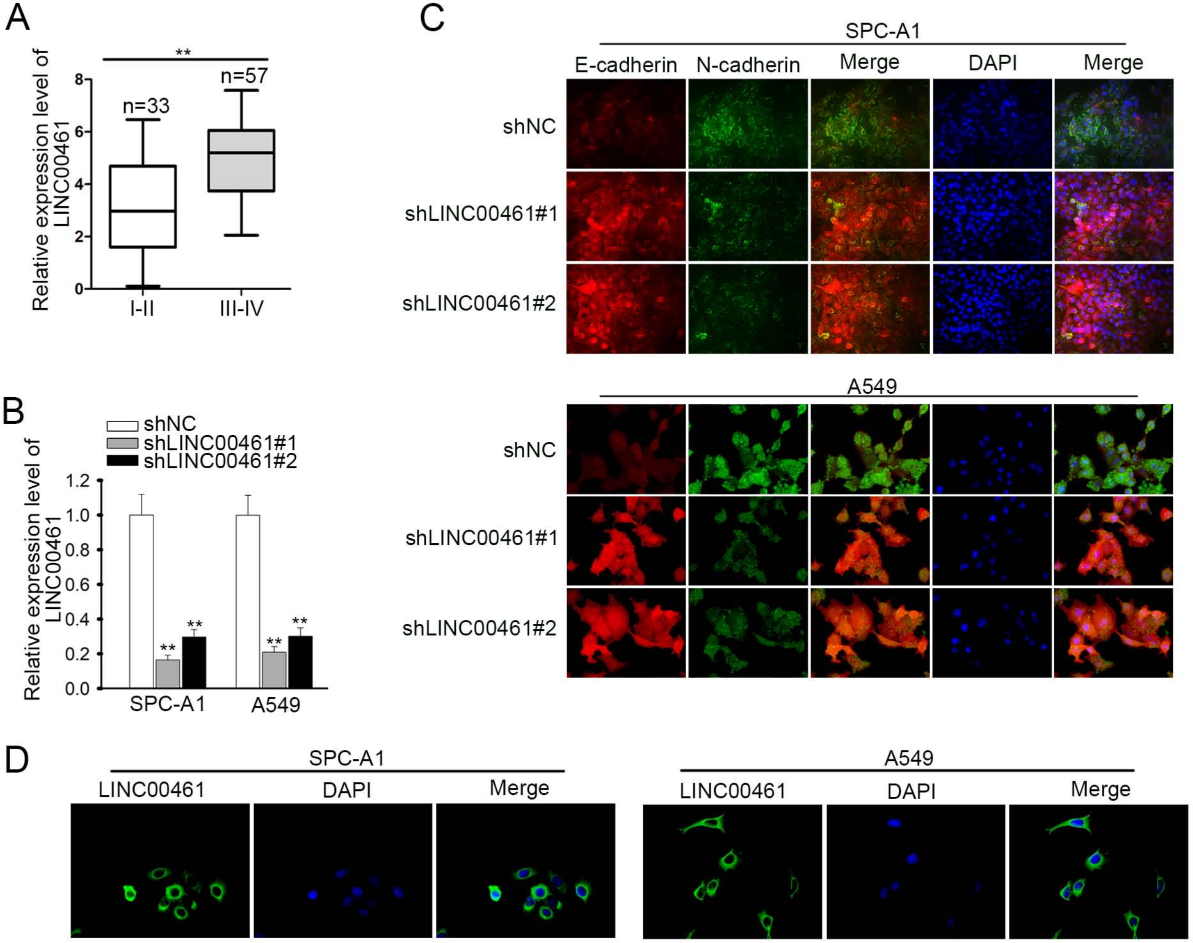

A
Relative expression level of LINC00461
**
n=33
n=57
8
6
4
2
0
I-II
III-IV
B
shNC
shLINC00461#1
shLINC00461#2
Relative expression level of LINC00461
1.2
1.0
0.8
0.6
0.4
0.2
0.0
**
SPC-A1
A549
C
SPC-A1
E-cadherin
N-cadherin
Merge
DAPI
Merge
shNC
shLINC00461#1
shLINC00461#2
A549
D
SPC-A1
LINC00461
DAPI
Merge
A549

Figure S1: (A) LINC00461 expressions inNSCLC tissues at I-II and III-IV stages. (B) Knockdown of LINC00461 was conducted via shRNAs targeting LINC00461. (C) Effect of shLINC00461 on EMT process was evaluated via immunofluorescence. (D) FISH assay was performed to determine LINC00461 subcellular localization.$^{**}P < 0.01$.

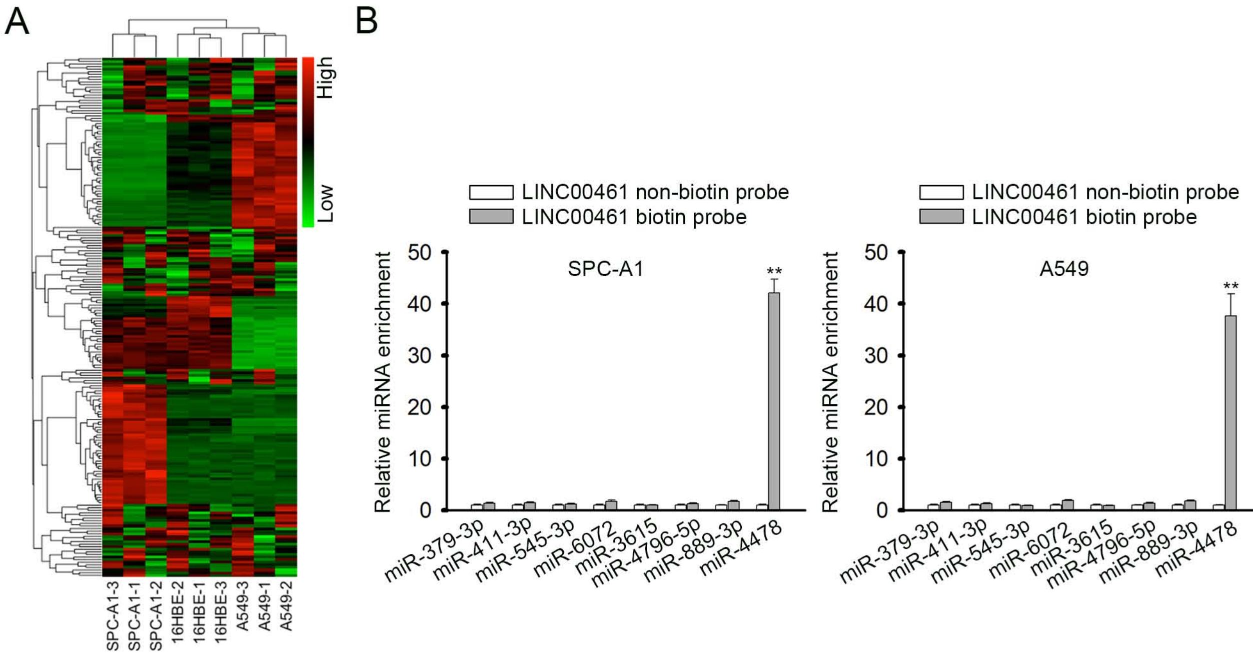
A
B
High
Low
SPC-A1-3
SPC-A1-1
SPC-A1-2
16HBE-2
16HBE-1
16HBE-3
A549-3
A549-1
A549-2
LINC00461 non-biotin probe
LINC00461 biotin probe
Relative miRNA enrichment
SPC-A1
A549
**
0
10
20
30
40
50
miR-379-3p
miR-411-3p
miR-545-3p
miR-6072
miR-3615
miR-4796-5p
miR-889-3p
miR-4478

Figure S2: (A) 200 miRNAs with higher LINC00461-binding score were obtained from DIANA-lncBase. RT-qPCR examined the expression of these miRNAs in NSCLC cell lines and 16HBE cell line. (B) RNA pull-down assay examined the interaction between LINC00461 and eight down-regulated miRNAs in NSCLC. $^{**}P < 0.01$.
